# Pre-transplant crossmatch-negative donor-specific anti-HLA antibody predicts acute antibody-mediated rejection but not long-term outcomes in kidney transplantation: an analysis of the Korean Organ Transplantation Registry

**DOI:** 10.3389/fimmu.2024.1420351

**Published:** 2024-07-11

**Authors:** Haeun Lee, Hanbi Lee, In O Sun, Jung Hwan Park, Jong-Won Park, Tae Hyun Ban, Jaeseok Yang, Myoung Soo Kim, Chul Woo Yang, Byung Ha Chung, Korean Organ Transplantation Registry Study Group

**Affiliations:** ^1^ Division of Nephrology, Department of Internal Medicine, Presbyterian Medical Center, Jeonju, Republic of Korea; ^2^ Division of Nephrology, Department of Internal Medicine, Seoul St. Mary’s Hospital, The Catholic University of Korea, Seoul, Republic of Korea; ^3^ Department of Nephrology, Konkuk University School of Medicine, Seoul, Republic of Korea; ^4^ Department of Nephrology, Yeungnam University Hospital, Daegu, Republic of Korea; ^5^ Division of Nephrology, Department of Internal Medicine, Eunpyeong St. Mary’s Hospital, The Catholic University of Korea, Seoul, Republic of Korea; ^6^ Division of Nephrology, Department of Internal Medicine, Yonsei University College of Medicine, Severance Hospital, Seoul, Republic of Korea; ^7^ Department of Surgery, Yonsei University College of Medicine, Seoul, Republic of Korea

**Keywords:** kidney transplantation, donor-specific anti-HLA antibody, solid phase assay, rejection, antibody-mediated rejection, desensitization

## Abstract

**Background:**

Pre-transplant donor-specific anti-human leukocyte antigen antibody (HLA-DSA) is a recognized risk factor for acute antibody-mediated rejection (ABMR) and allograft failure. However, the clinical relevance of pre-transplant crossmatch (XM)-negative HLA-DSA remains unclear.

**Methods:**

We investigated the effect of XM-negative HLA-DSA on post-transplant clinical outcomes using data from the Korean Organ Transplantation Registry (KOTRY). This study included 2019 living donor kidney transplant recipients from 40 transplant centers in South Korea: 237 with HLA-DSA and 1782 without HLA-DSA.

**Results:**

ABMR developed more frequently in patients with HLA-DSA than in those without (5.5% vs. 1.5%, p<0.0001). Multivariable analysis identified HLA-DSA as a significant risk factor for ABMR (odds ratio = 3.912, 95% confidence interval = 1.831–8.360; *p*<0.0001). Furthermore, the presence of multiple HLA-DSAs, carrying both class I and II HLA-DSAs, or having strong HLA-DSA were associated with an increased incidence of ABMR. However, HLA-DSA did not affect long-term clinical outcomes, such as allograft function and allograft survival, patient survival, and infection-free survival.

**Conclusion:**

Pre-transplant XM-negative HLA-DSA increased the risk of ABMR but did not affect long-term allograft outcomes. HLA-incompatible kidney transplantation in the context of XM-negative HLA-DSA appears to be feasible with careful monitoring and ensuring appropriate management of any occurrence of ABMR. Furthermore, considering the characteristics of pre-transplant XM-negative HLA-DSA, the development of a more detailed and standardized desensitization protocol is warranted.

## Introduction

Patel et al. initially reported a strong association between positive complement-dependent cytotoxicity crossmatch (CDC-XM) test and hyperacute rejection (1). The presence of pre-transplant donor-specific anti-human leukocyte antigen antibody (HLA-DSA) is widely recognized as a major cause of acute antibody-mediated rejection (ABMR) and subsequent allograft failure (2). Technical developments in diagnostic immunology have led to increased sensitivity and specificity in the detection of HLA-DSA. HLA-DSA that may not have been identified using cell-based assays, such as CDC-XM or flow cytometry crossmatch (FCXM), can now be confirmed using solid phase assays (SPA) (3).

Currently, there is consensus regarding the incorporation of SPA in addition to crossmatch (XM) tests for immunological risk assessment prior to kidney transplantation (KT) (4). The SPA has several advantages over other cell-based assays. Unlike cell-based assays, SPA can discriminate immunologically relevant HLA-DSAs from false-positive results because it is not affected by autoantibodies or non-HLA antibodies (5). Furthermore, SPA is qualitative, enable precise identification of HLA antibodies, and provides semi-quantitative mean fluorescence intensity (MFI) values. Assessing the HLA-DSA strength presented by MFI values has made it possible to predict the occurrence of post-transplant ABMR and allograft survival in more detail (6). Additionally, desensitization protocols based on monitoring MFI values have been proposed (7–9).

However, previous studies investigating the effect of pre-transplant HLA-DSA in patients with negative XM tests have shown conflicting results (10, 11). The cutoff MFI value of HLA-DSA varies across centers, and there is also variation in the choice of induction and maintenance immunosuppressive regimens, making standardized comparisons challenging (10). Therefore, each country and center has different protocols for managing XM-negative HLA-DSA before transplantation, including decisions on whether to proceed with transplantation, perform desensitization, and the level of desensitization required. In a previous single-center study, we reported that pre-transplant XM-negative HLA-DSA increased the incidence of ABMR without increasing allograft failure or patient death (12). In the present study, we investigated the effect of XM-negative HLA-DSA on post-transplant clinical outcomes using a nationwide multicenter cohort from the Korean Organ Transplantation Registry (KOTRY).

## Methods

### Study population

**Figure 1:**
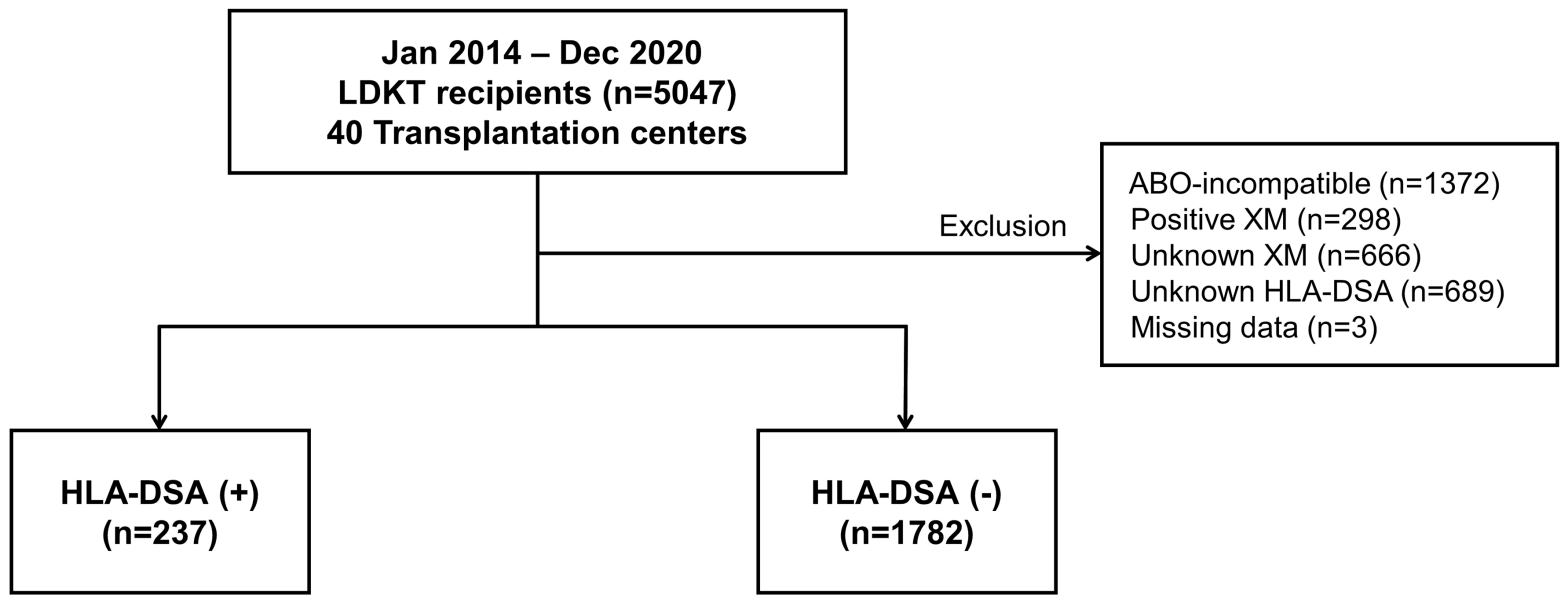
Distribution of patient population based on the presence of pre-transplant HLA-DSA. HLA-DSA, donor-specific anti-human leukocyte antigen antibody; LDKT, living donor kidney transplantation; XM, crossmatch.

KOTRY is a nationwide transplant registry prospectively collected since 2014 by the Korean Society for Transplantation (13, 14). We analyzed data from the KOTRY, including 5047 cases of living donor KT (LDKT) performed between January 2014 and December 2020 at 40 kidney transplantation centers. Patients who had undergone ABO-incompatible transplantation, tested positive for XM, did not have available XM or HLA-DSA results, or were missing essential data were excluded from the study. Finally, 2019 patients were eligible for the analysis. The patients were divided into two groups: HLA-DSA-positive (n=237) and HLA-DSA-negative (n=1782) (**Figure 1**). The mean follow-up period of this study was 20.5 months.

### Immunologic workup and detection of HLA-DSA

Immunological workups were performed according to the protocols of each center, but generally followed consensus practices. Our center’s detailed pre-transplant immunologic workup protocol, which is representative of practices used across the cohort, has been reported previously (7). As a baseline immunologic test, panel reactive antibody (PRA)-Luminex screening and XM tests, using both CDC-XM and FCXM, were performed in all patients. In patients with a PRA-Luminex screening results of ≥20%, or a positive result of XM test, the presence of HLA-DSA was investigated using the Luminex single antigen (LSA) assay.

PRA-Luminex screening test was done by the Luminex method (Lifecodes LifeScreen Deluxe kits; Hologic Gen-Probe Inc., San Diego, CA, USA) and was presented as %PRA. LSA assay for HLA-DSA was performed according to the manufacturer’s instructions, using Lifecodes LifeScreen Deluxe kits (Tepnel Lifecodes Corp., Stamford, CT). Briefly, microbeads coated with purified HLA class I/class II glycoproteins were incubated with 12.5 μL of patient serum in 96-well plates for 30 minutes. After three washes with a vacuum manifold, the beads were incubated with 50 μL of a 1:10 dilution of R-phycoerythrin (PE)-conjugated goat anti-human IgG for 30 minutes. After washing, the test samples were analyzed using the Quick-Type User’s Manual Research Use Only program, version 2.4, of the LABScan100 flow cytometer (Luminex Corp, Austin, TX); both positive and negative controls were included. The positive criterion was an MFI value of >1000.

In all recipients and donors, HLA typing was performed by the DNA molecular typing method, using reverse sequence-specific oligonucleotide probes and the RELI TM SSO HLA-A,B,Cw,DR,DQ Typing Kit (Dynal Biotech Ltd., Bromborough, UK). If the LSA assay-detected anti-HLA antibody in the patient corresponded to the HLA type of the donor, it was classified as a HLA-DSA. The results were presented as MFI values, and patients were classified into four groups based on their peak level at baseline: strong (>10000), moderate (5000–10000), weak (1000–5000), and negative (<1000). If two or more HLA-DSAs were detected in a single patient’s serum, the peak MFI (MFI^peak^) value was defined as that of the HLA-DSA with the strongest reactivity. The MFI values refer to the last pre-transplant serum. In cases where multiple measurements were taken, we recorded the MFI^peak^ value. For patients who underwent desensitization therapy, we used the MFI^peak^ value recorded before the desensitization.

### Crossmatch tests

The methods for the CDC-XM and FCXM have been previously described (15). For the CDC-XM, donor T-and B-cells were isolated using CD19 monoclonal antibody attached to beads. One microliter of donor cell suspension (2 × 10^6 cells/mL) was incubated with 1 μL of recipient serum for 30 minutes at room temperature. Anti-human globulin (1 μL) was then added and incubated for 30 minutes at 37°C. After washing the cells, rabbit complement was added and incubated for 60 minutes at room temperature. The cells were stained with acridine orange and ethidium bromide and observed for cytotoxicity using an immunofluorescent microscope. CDC results for T- and B-cells were considered positive when cell death exceeded that of the negative control well by 20%.

For the FCXM, 2 × 10^5 donor lymphocytes were added to 50 μL of patient serum and then incubated for 30 minutes at room temperature. Fluorescein isothiocyanate-labeled anti-human IgG (DAKO, Tokyo, Japan) and phycoerythrin-labeled CD19 or CD3 (DAKO) were added and incubated for 30 minutes. After washing the cells, a Coulter EPICS XL (Beckman Coulter, San Diego, CA, USA) was used for analysis. A positive FCXM was defined as a displacement of the mean channel fluorescence (MCF) by more than 10 channels relative to a negative control and donor autologous control. We also confirmed positive cases as having a relative median fluorescence (test MCF ÷ [recipient autologous MCF + donor autologous MCF + healthy autologous MCF]/3) ≥ 1.5 and a test MCF greater than that of the negative MCF + 3SDs.

### Definition of XM-negative HLA-DSA

An XM test was conducted between the donor and recipient prior to transplantation. The results were recorded separately for T-cell and B-cell CDC-XM (T-/B-CDC-XM) and T-cell- and B-cell FCXM (T-/B-FCXM). XM negativity was confirmed when both T-/B-CDC-XM and T-/B-FCXM results showed no reactivity. Patients missing any of T/-B-CDC-XM or T-/B-FCXM were excluded from the analysis. However, if both T-FCXM and B-FCXM results were confirmed negative, cases with missing T-CDC-XM or B-CDC-XM values were assumed to be negative and included in the study. Since 2017, the persistence of HLA-DSA has been monitored in patients with pre-transplant HLA-DSA at 6 and 12 months after transplantation and annually thereafter.

### Desensitization protocol

Desensitization protocols for pre-transplant HLA-DSA have been previously reported (7, 8, 16–19). Most centers implemented similar protocols for recipients with positive XM before transplantation. Briefly, these protocols included RTX, PP with or without low-dose intravenous immunoglobulin (IVIG). A single dose of RTX was administered one week to one month before transplantation, with dosages ranging from 100 mg to 375 mg/m². PP was performed using 5% albumin or fresh-frozen plasma, with the number of sessions determined by the MFI value of the HLA-DSA. IVIG was administered at a dose of 0.1 g/kg after each PP session. In some centers, Bortezomib (a proteasome inhibitor) was additionally used at 1.3mg/m² for four doses if the patient did not respond to desensitization using RTX and PP. The goal of desensitization for XM-positive patients was to achieve a negative conversion in the XM test and reduce the MFI value of HLA-DSA to below 3000 or 5000 before transplantation. XM tests and the MFI values of HLA-DSAs were monitored according to a scheduled follow-up during the desensitization period to assess treatment effectiveness.

However, detailed reports on desensitization protocols for patients with pre-transplant XM-negative HLA-DSA were scarce. Our previous study (7) described a protocol where patients with weak XM-negative HLA-DSA (MFI value <5000) received RTX at a dose of 375mg/m² 7–10 days before transplantation, only if PRA screening results were > 50%. Most centers, however, performed desensitization similar to XM-positive protocols, tailored to the patient’s immunologic risk, general medical condition, infection risk, and morbidity.

### Clinical outcomes

The primary outcome of this study was the incidence of biopsy-proven allograft rejection (BPAR). Patients with clinically diagnosed rejections that were not confirmed through biopsy were excluded. BPAR was diagnosed and classified as T-cell-mediated rejection (TCMR) and ABMR by the Banff classification at the time of biopsy (20). Considering the limited follow-up duration, acute rejections were analyzed, whereas chronic rejections (14 cases of chronic TCMR and 8 cases of chronic ABMR) were excluded. Borderline changes were not considered as BPAR. Mixed rejections were not differentiated and were included with acute rejection events encompassing both TCMR and ABMR. The secondary outcomes included changes in allograft function, allograft survival, patient survival, and infection-free survival. Serum creatinine levels were collected at discharge, 6 and 12 months after KT, and annually. The estimated glomerular filtration rate (eGFR) was calculated using the Chronic Kidney Disease Epidemiology Collaboration (CKD-EPI) equation (21) at each time point, excluding patients with failed grafts. Graft failure was defined as return to maintenance dialysis or receipt of a new KT. The missing eGFR values of these patients were not imputed. The comparison of eGFR focused on patients with maintained allograft function. Because the number of patients who experienced graft failure was small, excluding these patients does not significantly reduce the sample size or the statistical power. For the analysis of death-censored graft failure, patients who died with a functional graft were censored at the date of death. An infectious episode was defined as an infectious event that required hospitalization. Infection-free graft survival was defined as the time from transplantation to the first infectious episode.

### Statistical analysis

Continuous variables are presented as the mean ± standard deviation or the median with interquartile range, depending on their distribution. Categorical variables are presented as frequencies and percentages (%). Student’s t-test or Mann-Whitney U test was used to analyze continuous variables. The chi-square test or Fisher’s exact test was used to compare categorical variables. Logistic regression analysis was used to explore predictors of ABMR. Clinical parameters that showed significant differences (*p-*value<0.1) in the univariate analysis or were known to cause ABMR were included in the initial multivariable model. A backward selection procedure was then implemented with a threshold of *p*<0.1 for inclusion and a *p*<0.05 being defined as statistically significant in the final model. A linear mixed model was employed to compare the changes in allograft function over time. Survival curves were generated using the Kaplan-Meier method and compared using the log-rank test. Survival outcomes, including death-censored graft survival, patient survival, and infection-free survival, were analyzed using univariate and multivariable Cox regression. Univariate Cox regression was performed to identify variables associated with the survival outcomes. Variables with a *p*<0.1 in the univariate analysis were included in the initial multivariable model, along with HLA-DSA to assess its impact on survival outcomes. A backward selection procedure was applied with a threshold of *p*<0.1 for inclusion and *p*<0.05 was defined as a statistically significant in the final model. For death-censored graft survival and infection-free survival, Fine-Gray regression was used to treat patient death as a competing event. All missing data were censored at the last follow-up. All statistical analyses were performed using R software (version 4.2.2; R Foundation for Statistical Computing, Vienna, Austria). Data visualization and additional analyses were performed using RStudio (version 2023.12.1 + 402; RStudio, PBC, Boston, MA) and GraphPad Prism (version 3.10; GraphPad Software, Inc., San Diego, CA, USA). Significance was set at *p*<0.05 (two-tailed).

## Results

### Baseline characteristics

The baseline characteristics of patients according to the presence of pre-transplant HLA-DSA are shown in **Table 1**. The mean age was higher and the dialysis vintage was longer in patients with HLA-DSA than in those without HLA-DSA. Patients with HLA-DSA were more often female (61.2% vs. 38.3%), underwent re-transplantation (9.7% vs. 5.6%), and had higher PRA levels. Primary renal disease (PRD), recipient or donor BMI, donor age, and cold ischemic time did not differ between the groups. The proportion of patients receiving anti-thymocyte globulin (ATG) as induction therapy was higher in the HLA-DSA (+) group than in the HLA-DSA (-) group (38.4% vs. 5.4%). In addition, patients with HLA-DSA underwent desensitization more frequently than those without HLA-DSA (40.5% vs. 1.9%). Several desensitization therapies have been used for patients with HLA-DSA: 32/96 were administered rituximab (RTX) plus plasmapheresis (PP) with or without IVIG, 20/96 received only RTX, and 5/96 received PP with or without IVIG. Desensitization was performed even in 33 patients without HLA-DSA. Among these, 28 patients underwent desensitization due to high pre-transplant PRA levels, 3 patients to prevent post-transplant recurrence of focal segmental glomerulosclerosis, one patient due to pre-transplant immune thrombocytopenia (for which PP with IVIG was performed), and in one patient, the reason for desensitization could not be determined from the available records. Most patients received tacrolimus as the main maintenance immunosuppressive agent, and this did not differ between the groups.

**Table 1:** Baseline characteristics stratified by the presence of pre-transplant HLA-DSA.

	HLA-DSA (+) (n=237)	HLA-DSA (-) (n=1782)	*p*-value
Recipient			
Age (years)	49.6 ± 11.5	47.7 ± 12.2	0.016
Female sex (n, %)	145 (61.2%)	683 (38.3%)	<0.0001
PRD (n, %)			0.125
Diabetes	58 (24.5%)	445 (25.0%)	
Hypertension	29 (12.2%)	252 (14.1%)	
Glomerulonephritis	74 (31.2%)	602 (33.8%)	
Polycystic kidney disease	18 (7.6%)	79 (4.4%)	
Others	5 (2.1%)	70 (3.9%)	
Unknown	53 (22.4%)	334 (18.7%)	
BMI	23.1 ± 3.7	23.2 ± 4.9	0.898
PRA class I (%)	19.0 (0–52.5)	0 (0–4.0)	<0.0001
PRA class II (%)	20.0 (0–50.5)	0 (0–2.0)	<0.0001
Dialysis vintage (months)	28.2 ± 54.6	19.1 ± 43.7	0.004
Follow up duration (months)	22.4 ± 19.4	20.3 ± 18.7	0.051
Donor			
Age (years)	46.9 ± 12.2	46.9 ± 11.8	0.745
Female sex (n, %)	115 (48.5%)	1042 (58.5%)	0.005
BMI	24.2 ± 3.1	24.3 ± 3.2	0.935
Transplant			
Re-transplant (n, %)	23 (9.7%)	100 (5.6%)	0.020
Cold ischemic time	56.4 ± 33.8	55.0 ± 32.5	0.769
Induction therapy (n, %)			<0.0001
No	2 (0.8%)	11 (0.6%)	
ATG	91 (38.4%)	96 (5.4%)	
Basiliximab	136 (57.4%)	1657 (93.0%)	
ATG and Basiliximab	8 (3.4%)	18 (1.0%)	
Maintenance immunosuppressant (n, %)			0.850
Tacrolimus	229 (96.6%)	1697 (95.2%)	
Cyclosporine	8 (3.4%)	79 (4.4%)	
Tacrolimus and Sirolimus	1 (0.4%)	9 (0.5%)	
Desensitization	96 (40.5%)	33 (1.9%)	<0.0001
RTX + PP ± IVIG	32 (13.5%)	2 (0.1%)	
RTX only	20 (8.4%)	11 (0.6%)	
PP ± IVIG	5 (2.1%)	5 (0.3%)	
Not recorded	39 (16.5%)	15 (0.8%)	

ATG, anti-thymocyte globulin; BMI, body mass index; HLA-DSA, donor-specific anti-human leukocyte antigen antibody; IVIG, intravenous immunoglobulin; PP, plasmapheresis; PRA, panel-reactive antibody; PRD, primary renal disease; RTX, rituximab.

### Characteristics of HLA-DSA

**Table 2** summarizes the characteristics of HLA-DSA. Of the 237 patients with HLA-DSAs, 73.8% had a single HLA-DSA, 21.1% had two distinct HLA-DSAs, and only 5.1% had three or more HLA-DSAs. According to the HLA-DSA class, 41.8% had class I HLA-DSA, 45.6% had class II HLA-DSA, and 12.7% had both class I and II HLA-DSA. The median value of MFI^peak^ was 2170 (interquartile range: 1400–3830) and the median value of total MFI of all HLA-DSAs (MFI^sum^) was 2540 (interquartile range: 1480–4670). The MFI values for class I and class II HLA-DSAs did not differ, with a median of 2200 (1430–3910) for class I and 2130 (1390–3710) for class II. Most HLA-DSAs (83.1%) were of weak intensity, 11.8% were moderate, and 4.2% were strong. Only 66 (27.9%) patients had their HLA-DSA measured 6 months after transplantation, and of them, HLA-DSA persisted in 39 patients (59.1%) and disappeared in 27 (40.9%). Among the 197 patients with weak HLA-DSA, 34 (17.3%) had persistent HLA-DSA, 22 (11.2%) had disappeared HLA-DSA, and 141 (71.6%) did not have follow-up measurements. Most of the disappeared HLA-DSA cases were of weak intensity, with 22 out of 27 classified in this category.

**Table 2:** Characteristics of pre-transplant HLA-DSA.

	HLA-DSA (+) (n=237)
HLA-DSA number	
1	175 (73.8%)
2	50 (21.1%)
3	9 (3.8%)
4	3 (1.3%)
HLA-DSA class	
Class I	99 (41.8%)
Class II	108 (45.6%)
Class I + II	30 (12.7%)
MFI^peak^	2170 (1400–3830)
MFI^peak^, group	
Weak (<5000)	197 (83.1%)
Moderate (5000–10000)	28 (11.8%)
Strong (>10000)	10 (4.2%)
MFI^sum^	2540 (1480–4670)
HLA-DSA at 6 months after KT	
Persistent	39 (16.5%)
Undetectable	27 (11.4%)
Not checked	171 (72.2%)

HLA-DSA, donor-specific anti-human leukocyte antigen antibody; MFI, mean fluorescence intensity; MFI^peak^, peak MFI; MFI^sum^, total MFI of all HLA-DSAs.

**Figure 2:**
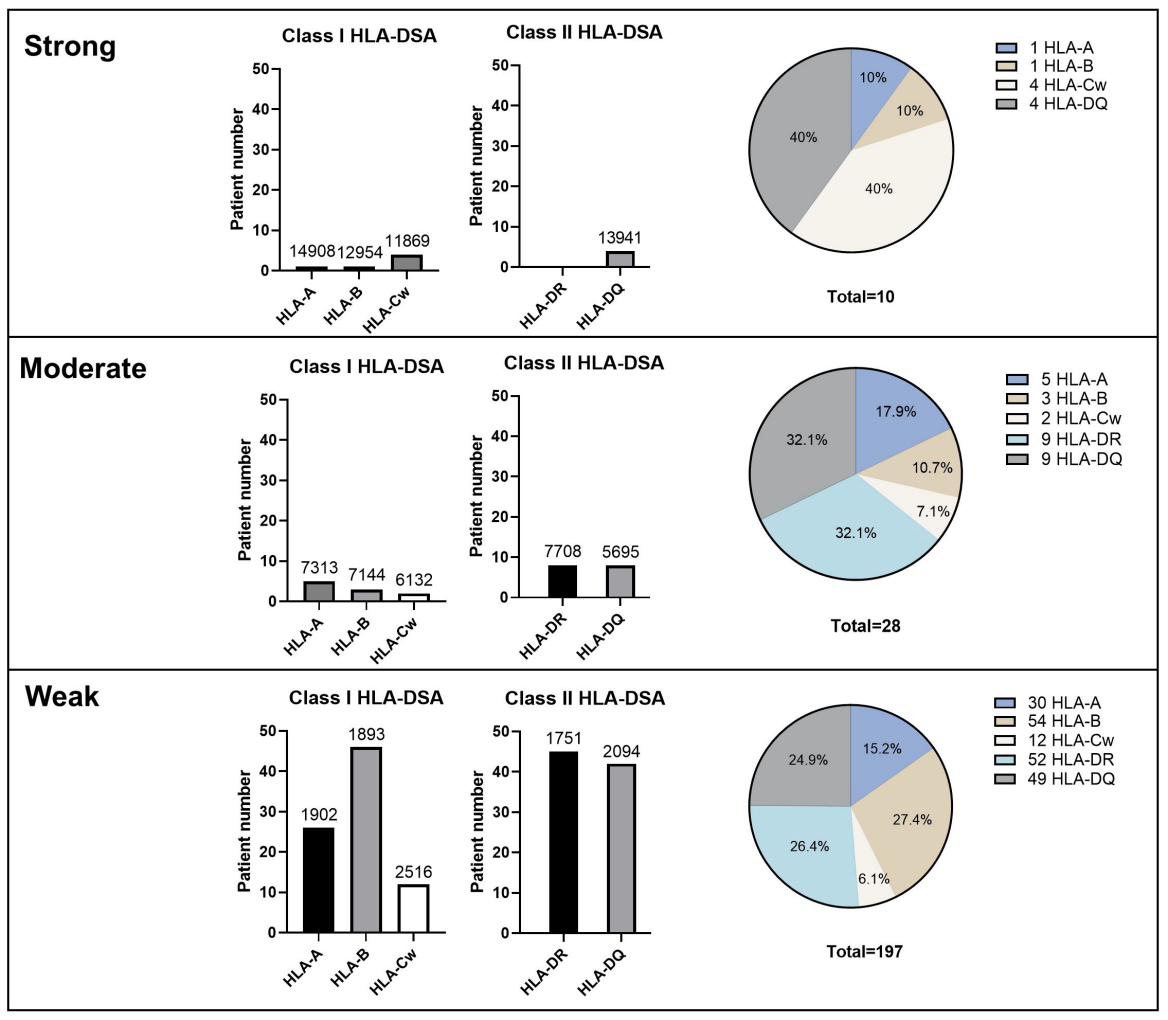
Immunologic characteristics of HLA-DSA according to the strength, class, and specificities of HLA-DSA. Numbers above the bar graphs mean median HLA-DSA MFI values. Pie graphs represent the percentage of HLA-DSA specificity within each HLA-DSA group classified by the peak MFI value. HLA-DSA, donor-specific anti-human leukocyte antigen antibody.

**Figure 2** illustrates the distribution of immunodominant HLA-DSA according to strength and specificity. Data for MFI values were unavailable for two patients. Overall, 114 (48.1%) patients had class I HLA-DSA and 123 (51.9%) had class II HLA-DSA as their immunodominant HLA-DSA. This even distribution between class I and class II HLA-DSAs was consistently observed across strong, moderate, and weak HLA-DSA groups. The majority of strong HLA-DSA consisted of anti-HLA-Cw (40%) and anti-HLA-DQ (40%) antibodies, although no significant difference was found. Distinctively, 11.1% of anti-HLA-Cw antibodies (2/18) and 6.5% of anti-HLA-DQ antibodies (4/62) were identified as strong HLA-DSA, which was higher than that of other HLA-DSA categories. In contrast, no strong HLA-DSA was detected among the anti-HLA-DR antibodies.

### Biopsy-proven allograft rejection

**Figure 3:**
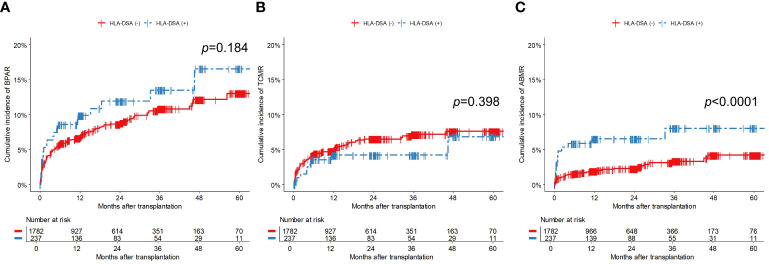
Cumulative incidence of **(A)** BPAR, **(B)** TCMR, and **(C)** ABMR. The numbers below the figures denote the numbers of KT recipients at risk in each subgroup. ABMR, antibody-mediated rejection; BPAR, biopsy-proven allograft rejection; HLA-DSA, donor-specific anti-human leukocyte antigen antibody; TCMR, T-cell- mediated rejection.

**Figure 3** shows the cumulative incidences of BPAR, TCMR, and ABMR. Most BPAR events occurred within the first year of transplantation (78.1%, 114/146). Specifically, the majority of TCMR (81.9%, 77/94) and ABMR (76.9%, 40/52) events occurred within the first year. During the first year, ABMR developed more frequently in the HLA-DSA (+) group than in the HLA-DSA (-) group (5.5%, 13/237 vs. 1.5%, 27/1782, *p*<0.0001). However, no significant difference in the incidences of BPAR (8.0%, 19/237 vs. 5.3%, 95/1782, *p*=0.125) and TCMR (3.4%, 8/237 vs. 3.9%, 69/1782, *p*=0.846) were found between the HLA-DSA (+) and HLA-DSA (-) groups. Univariate and multivariable analyses were conducted to identify the correlations between the variables and occurrence of ABMR (**Table 3**). Multivariable analysis revealed that pre-transplant HLA-DSA was an independent predictor of ABMR, with an odds ratio (OR) of 3.912 (95% confidence interval [CI]: 1.831–8.360, *p*<0.0001). Treatment for ABMR ranged from observation to the use of multiple therapies, including steroid pulse therapy, PP ± IVIG, RTX, bortezomib, and their various combinations (**Supplementary Table S1**). Treatment decision was based on the individual patient’s medical condition. Five patients were managed with observation, and nine received steroid pulse therapy with or without immunosuppressant adjustment. Seven patients received treatment based on PP ± IVIG. Eighteen patients received RTX and one patient received bortezomib in addition to the aforementioned therapies.

**Table 3:** Prediction of ABMR within the first year of transplantation.

Variables	Univariate		Multivariable	
	Odds ratio (95% CI)	*p*-value	Odds ratio (95% CI)	*p*-value
HLA-DSA	3.772 (1.919–7.417)	<0.0001	3.912 (1.831–8.360)	<0.0001
Patient female sex	1.605 (0.857–3.005)	0.139		
Patient age	0.986 (0.961–1.011)	0.268	0.983 (0.958–1.008)	0.182
Dialysis vintage	0.997 (0.988–1.006)	0.468		
Donor age	1.013 (0.986–1.041)	0.348	1.012 (0.984–1.040)	0.418
Cold ischemic time	0.998 (0.988–1.009)	0.728		
ATG (vs Basiliximab)	1.824 (0.753–4.421)	0.183		
ATG and Basiliximab (vs Basiliximab)	4.586 (1.039–20.232)	0.044		
Tacrolimus (vs CsA)	1.731 (0.235–12.756)	0.590		

ABMR, antibody-mediated rejection; ATG, anti-thymocyte globulin; BMI, body mass index; CI, confidence interval; CsA, cyclosporine; HLA-DSA, donor-specific anti-human leukocyte antigen antibody.

After treatment for ABMR, among 13 patients with HLA-DSA, 8 demonstrated resolution with stabilized allograft function. Four patients demonstrated resolution; however, they did not recover to baseline allograft function, with serum creatinine levels < 2.8 mg/dL. One patient exhibited an inadequate response, resulting in allograft failure within one month. Among 27 patients without HLA-DSA, 20 demonstrated resolution with stabilized allograft function. Four patients demonstrated resolution but did not recover to baseline allograft function, with serum creatinine levels < 2.8 mg/dL. Three patients demonstrated resolution but continued to experience allograft dysfunction, with serum creatinine levels ≥ 2.8 mg/dL. No patient experienced allograft failure within one month due to ABMR. Of the 40 patients diagnosed with ABMR within the first year, 4 experienced death-censored allograft failure: 1 of the 13 with HLA-DSA and 3 of the 27 without HLA-DSA.

The univariate logistic regression model presented in **Table 4** illustrates the correlation between HLA-DSA characteristics and the development of ABMR. ABMR developed in 3.4% (6/175) of patients with a single HLA-DSA, 12.0% (6/50) of those with two HLA-DSAs, and 11.1% (1/9) of those with three HLA-DSAs. The risk of ABMR was higher in patients with multiple HLA-DSAs compared to those with a single HLA-DSA, with ORs of 2.310 for n=1, 8.860 for n=2, and 8.120 for n=3. When comparing patients with two HLA-DSAs based on the strength of the HLA-DSA, ABMR developed in 5.3% (2/38) of those with weak HLA-DSAs, 33.3% (3/9) of those with moderate HLA-DSAs, and 33.3% (1/3) of those with strong HLA-DSAs. With the same number of two HLA-DSAs, the risk of ABMR was higher in patients with stronger HLA-DSAs (OR=3.610 for weak, OR=32.500 for moderate, OR=32.500 for strong). Furthermore, patients with both class I and II HLA-DSAs exhibited a higher incidence of ABMR compared to those with only one class of HLA-DSA. ABMR developed in 4.0% (4/99) of patients with class I HLA-DSAs, 4.6% (5/108) of those with class II HLA-DSAs, and 13.3% (4/30) of those with both class I and class II HLA-DSAs. The risk of ABMR was higher in patients with both class I and II HLA-DSAs (OR=10.000) than those with only one class of HLA-DSA (OR=2.740 for class I; OR=3.160 for class II). In addition, a higher incidence of ABMR was observed in patients with moderate (14.3%, 4/28) or strong (10.0%, 1/10) HLA-DSAs compared to those with weak HLA-DSA (4.1%, 8/197). The risk of ABMR was higher in patients with moderate or strong HLA-DSAs compared to those with weak HLA-DSAs, with ORs of 2.750 for weak, 10.850 for moderate, 7.230 for strong HLA-DSA. In the receiver operating characteristics (ROC) curve analysis using the MFI^peak^ and MFI^sum^, the area under the ROC curve (AUC) values were 0.622 (95% CI: 0.427–0.818) for MFI^peak^ and 0.622 (95% CI: 0.425–0.820) for MFI^sum^, respectively (**Supplementary Figure S1**). The optimal cut-off values for predicting ABMR were determined to be 5362 for MFI^peak^ and 10724 for MFI^sum^.

**Table 4:** Prediction of ABMR based on the characteristics of HLA-DSA.

Variables	ABMR (n/total)	Odds ratio (95% CI)	*p*-value
number = 1	6/175	2.310 (0.940–5.670)	0.068
number = 2	6/50	8.860 (3.480–22.550)	<0.0001
- MFI^peak^<5000 (weak)	2/38	3.610 (0.830–15.760)	0.088
- 5000<MFI^peak^ <10000 (moderate)	3/9	32.500 (7.720–136.770)	<0.0001
- MFI^peak^ >10000 (strong)	1/3	32.500 (2.860–369.300)	<0.0001
number = 3	1/9	8.120 (0.980–67.240)	0.052
number = 4	0/3	N.A.	N.A.
Class I	4/99	2.740 (0.940–7.980)	0.065
Class II	5/108	3.160 (1.190–8.360)	0.021
Class I+II	4/30	10.000 (3.270–30.620)	0.0001
MFI^peak^ <5000 (weak)	8/197	2.750 (1.230–6.150)	0.013
5000<MFI^peak^ <10000 (moderate)	4/28	10.850 (3.520–33.400)	<0.0001
MFI^peak^ >10000 (strong)	1/10	7.230 (0.880–59.080)	0.065

ABMR, antibody-mediated rejection; ATG, anti-thymocyte globulin; CI, confidence interval; HLA-DSA, donor-specific anti-human leukocyte antigen antibody; MFI, mean fluorescence intensity; MFI^peak^, peak MFI; N.A., not applicable.

### Changes in allograft function, allograft survival, and patient survival

**Figure 4:**
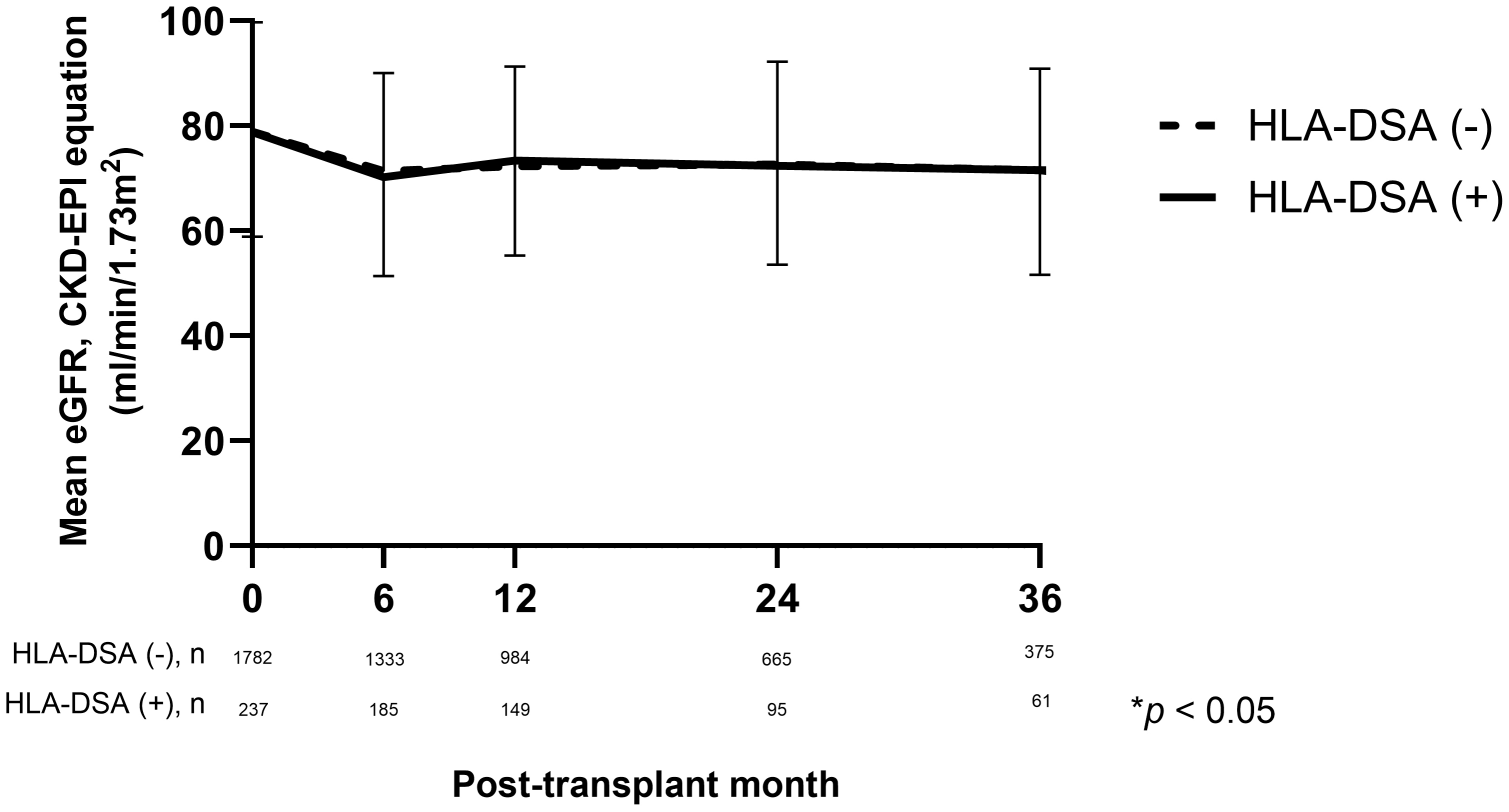
Comparison of kidney allograft function based on eGFR using CKD-EPI equation (mL/min/1.73m^2^) according to the presence of pre-transplant HLA-DSA. CKD-EPI, Chronic Kidney Disease-Epidemiology Collaboration; eGFR, estimated glomerular filtration rate; HLA-DSA, donor-specific anti-human leukocyte antigen antibody.

At the time of discharge, the mean eGFR was 78.8 ml/min/1.73 m^2^ in the HLA-DSA (+) group and 78.9 ml/min/1.73 m^2^ in the HLA-DSA (-) group. After 6 months of transplantation, the eGFR decreased to 70.2 ml/min/1.73 m^2^ in the HLA-DSA (+) group and 71.4 ml/min/1.73 m^2^ in the HLA-DSA (-) group. During the follow-up period, the eGFR values recovered 12 months post-transplantion, reaching 73.4 ml/min/1.73 m^2^ in the HLA-DSA (+) group and 72.3 ml/min/1.73 m^2^ in the HLA-DSA (-) group. No difference in the change in allograft function was found between the two groups until 36 months post-transplantation (**Figure 4**).

**Figure 5:**
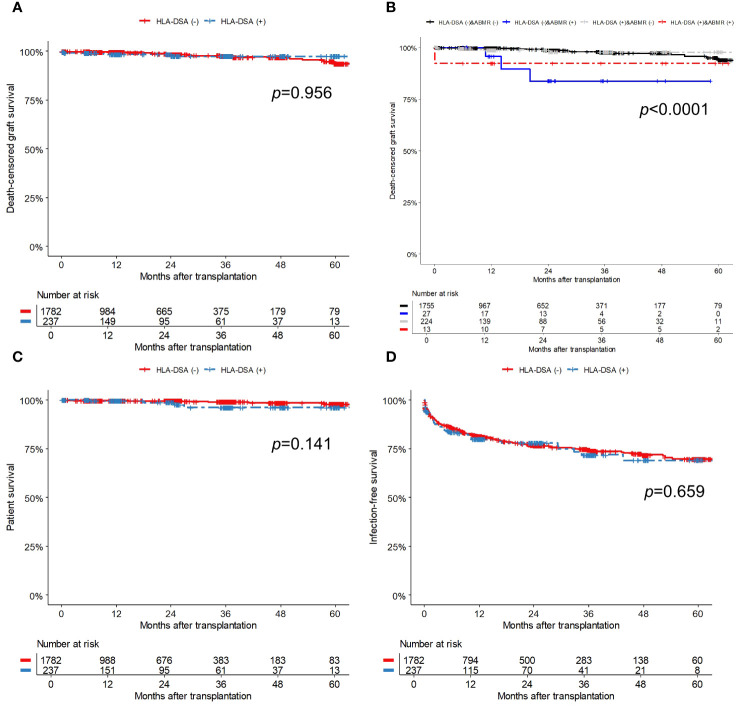
Comparison of survival outcomes; **(A)** Death-censored graft survival based on the presence of pre-transplant HLA-DSA, **(B)** Death-censored graft survival based on the presence of pre-transplant HLA-DSA and ABMR, **(C)** Patient survival, and **(D)** Infection-free survival. The numbers below the figures denote the numbers of KT recipients at risk in each subgroup. ABMR, antibody-mediated rejection; HLA-DSA, donor-specific anti-human leukocyte antigen antibody.

During the follow-up period, 30 (1.5%) patients experienced death-censored graft failure. Allograft rejection was the most common cause of graft failures (30.0%, 10/30). Other causes included BK polyomavirus nephropathy (4/30, 13.3%), acute pyelonephritis (1/30, 3.3%), non-compliance (1/30, 3.3%), post-operative complication (1/30, 3.3%), primary graft failure (1/30, 3.3%), malignancy (1/30, 3.3%), and thrombotic microangiopathy (1/30, 3.3%). The incidence (per 1000 person-years) of death-censored graft failure was 9.1 for the HLA-DSA (+) group and 8.7 for the HLA-DSA (-) group. As presented in the Kaplan-Meier curve (**Figure 5A**), the death-censored allograft survival rate did not differ according to the presence of HLA-DSA. Notably, additional analysis using a Kaplan-Meier curve, which categorized patients into four groups based on the presence of HLA-DSA and ABMR, revealed that ABMR, regardless of HLA-DSA status, was associated with poor death-censored graft survival (**Figure 5B**).

Sixteen patients (0.8%) died during the follow-up, and of them, four had HLA-DSA while the remaining 12 did not. Among the patients with HLA-DSA, two died from infection, one by suicide, and one from traumatic intracerebral hemorrhage. Among the patients without HLA-DSA, three died from infection, three from sudden cardiac arrest, and two from malignancy; the cause of death in four patients remains unknown. The incidence rate (per 1000 person-years) of patient death was 9.0 for the HLA-DSA (+) group and 4.0 for the HLA-DSA (-) group. Patient survival did not differ between the groups (**Figure 5C**). In the univariate and multivariable Cox regression models (**Table 5**), the presence of pre-transplant HLA-DSA was not a significant risk factor for either death-censored graft failure or patient death.

**Table 5:** Univariate and multivariable Cox regression models.

Variables	Univariate Cox		Multivariable Cox	
For death-censored graft failure^*^	HR (95% CI)	*p*-value	HR (95% CI)	*p*-value
HLA-DSA	0.870 (0.260–2.960)	0.820		
Patient age	0.970 (0.940–1.000)	0.028	0.970 (0.940–1.000)	0.049
Patient BMI	1.030 (1.010–1.050)	0.005	1.020 (1.000–1.040)	0.019
Dialysis vintage	0.960 (0.920–1.000)	0.033	0.960 (0.920–1.000)	0.038
For patient death	HR (95% CI)	*p*-value	HR (95% CI)	*p*-value
HLA-DSA	2.284 (0.736–7.093)	0.153		
Patient age	1.081 (1.026–1.139)	0.003	1.086 (1.024–1.151)	0.006
Patient female sex	0.309 (0.087–1.093)	0.068	0.224 (0.050–1.006)	0.051
Cold ischemic time	1.006 (0.999–1.013)	0.087	1.005 (0.999–1.012)	0.118
For infection-free survival^*^	HR (95% CI)	*p*-value	HR (95% CI)	*p*-value
HLA-DSA	1.040 (0.770–1.420)	0.790		
Patient female sex	1.470 (1.200–1.800)	<0.0001	1.470 (1.200–1.810)	<0.0001
Donor age	1.010 (1.000–1.020)	0.047	1.010 (1.000–1.020)	0.074
PRD, hypertension (vs. diabetes)	0.590 (0.400–0.880)	0.010	0.590 (0.390–0.870)	0.009
PRD, Polycystic kidney disease (vs. diabetes)	1.510 (0.990–2.320)	0.056	1.410 (0.920–2.150)	0.120
ATG (vs. Basiliximab)	1.320 (0.950–1.830)	0.100		

ATG, anti-thymocyte globulin; BMI, body mass index; CI, confidence interval; HR, hazard ratio; HLA-DSA, donor-specific anti-human leukocyte antigen antibody; PRD, primary renal disease.

^*^ For death censored graft failure and infection-free survival, Fine and Gray’s regression was used, treating patient death as a competing event.

### Post-transplant infections

Post-transplant infections were observed in 375 (18.6%) patients during the follow-up period, resulting in 633 infectious events. The incidence rates (per 1000 person-years) was 187.0 for the HLA-DSA (+) group and 182.4 for the HLA-DSA (-) groups. **Table 6** provides detailed information on infectious episodes that occurred during the first year of transplantation, categorized by pathogen. No significant differences in the total infection rate or presence of specific pathogens were observed between the groups. Infection-free survival did not differ significantly between the groups (**Figure 5D**). In the univariate and multivariable Cox regression models (**Table 5**), patient female sex was a significant predictor of post-transplant infection, while patients with hypertension as their PRD had a lower risk of post-transplant infections compared to those with diabetes. Pre-transplant HLA-DSA did not increase infection risk.

**Table 6:** Infection episodes in the first year of transplantation.

	HLA-DSA (+) (n=237)	HLA-DSA (-) (n=2019)	*p*-value
**Infection, n(%)**	41 (17.3%)	269 (15.1%)	0.430
Bacterial	27 (11.4%)	164 (9.2%)	0.335
Viral	13 (5.5%)	97 (5.4%)	1.000
Fungal	2 (0.8%)	16 (0.9%)	1.000
Pneumocystis jirovecii	2 (0.8%)	18 (1.0%)	1.000
Mycobacterial	1 (0.4%)	4 (0.2%)	0.465
Unknown	4 (1.7%)	23 (1.3%)	0.549

HLA-DSA, donor-specific anti-human leukocyte antigen antibody.

### Desensitization and post-transplant clinical outcomes

Of the 237 patients in the HLA-DSA (+) group, 96 underwent desensitization therapy before transplantation while the remaining 141 did not. We performed subgroup analysis to compare whether desensitization therapy improved the post-transplant clinical outcomes in patients with pre-transplant HLA-DSA. Comparison of baseline characteristics between the two subgroups (**Supplementary Table S2**) showed that desensitized patients had a higher proportion of females (70.8% vs. 54.6%, *p*=0.017). Desensitized patients had higher PRA class I values (34.0% vs. 11.0%, *p*=0.038) and class II values (32.5% vs. 15.0%, *p*=0.028) compared to non-desensitized patients. While there was no difference in the HLA-DSA class or number, the HLA-DSA were stronger in desensitized patients than in non-desensitized patients (MFI^peak^, 2670 vs. 1970, *p*=0.007; MFI^sum^, 3270 vs. 2100, *p*=0.002).

Patients who underwent desensitization displayed a trend toward a higher incidence of BPAR and ABMR than those who did not receive desensitization therapy (**Supplementary Figure S2**), although the difference was not significant. To address the potential bias, we evaluated the development of BPAR in relation to desensitization and further stratified the results using HLA-DSA MFI values. Our findings revealed a trend toward a higher incidence of ABMR in desensitized patients, regardless of the MFI values. (**Supplementary Figure S3**). We also considered using propensity score methods; however, the limited sample size in both groups, along with numerous missing values for key matching variables, prevented the implementation of these methods. Allograft function (**Supplementary Figure S4**), as well as allograft and patient survival (**Supplementary Figures S5A**, **S5B**), showed no differences based on pre-transplant desensitization therapy. However, infection-free survival (**Supplementary Figure S5C**) tended to be lower in the desensitized group.

## Discussion

This study showed that pre-transplant XM-negative HLA-DSA increased the risk of acute ABMR. Furthermore, ABMR development was more prevalent when there were multiple HLA-DSAs, when both class I and II DSA were present, and with high HLA-DSA MFI values. However, XM-negative HLA-DSA did not significantly affect long-term clinical outcomes such as allograft function, allograft survival, or patient survival.

End-stage kidney disease (ESKD) is a rapidly increasing global health burden (22). The incidence of ESKD has increased substantially in East Asian countries, including South Korea. This increase is associated with an aging population and a high burden of metabolic diseases (23). Patients with ESKD require kidney replacement therapy. KT is known to provide long-term survival benefits compared to dialysis (24). However, several significant barriers to KT exist, including human leukocyte antigen (HLA) incompatibility. In the past, HLA incompatibility was considered a contraindication to KT, as confirmed by a positive CDC-XM test (1). With the sharp increase in the prevalence of ESKD and the limited availability of kidney donors, efforts have been made to overcome the challenge of HLA incompatibility. Desensitization is a representative method, and successful transplantation has been reported in HLA-incompatible KT cases after desensitization (25). With the introduction of a highly sensitive SPA method, accurate identification of HLA-DSAs has become possible, thereby enabling sophisticated risk stratification (3). The presence of pre-transplant HLA-DSA is associated with unfavorable clinical outcomes such as increased acute rejection, specifically in the form of ABMR and allograft failure (26). However, whether HLA-DSA that is undetected in cell-based assays and only detected in SPA has clinical significance is debatable (3, 10, 11). Furthermore, reports on desensitization in HLA-incompatible KT have predominantly aimed to achieve negative conversion in patients with positive XM results (27, 28). Data on the outcomes of desensitization for those with XM-negative HLA-DSAs are limited.

First, we analyzed the incidence of BPAR within 1 year after KT in relation to the presence of pre-transplant HLA-DSA. In our cohort, BPAR was reported in 8.0% of the DSA (+) group, and 5.3% of the DSA (-) group. ABMR developed in 5.5% of the DSA (+) group and 1.5% of the DSA (-) group. These incidence rates were lower than those reported in previous studies, including recent nationwide studies (29–31). Ziemann et al. (30) included ABO-incompatible KT in their analysis, and FCXM was not performed in most patients, which may have overestimated the impact of HLA-DSA on the development of ABMR. In a study by Wehmeier et al. (31), deceased donors (64%) were included more frequently than living donors (36%). Deceased donor kidneys may exhibit increased expression of adhesion molecules and HLA antigens compared with living donor kidneys (32), making them more immunogenic to HLA antigens. This may have led to a higher incidence of ABMR. Immunosuppressive protocols for KT change over time and vary depending on the country and center. In our cohort, 40.5% of patients in the HLA-DSA (+) group received desensitization therapy before transplantation. In addition, 99.2% (235/237) of the patients in the HLA-DSA (+) group underwent induction therapy, with 41.8% receiving either ATG or a combination of ATG and basiliximab. For maintenance immunosuppression, tacrolimus was included as the main drug in the regimen for 96.6% (229/237) of patients. Compared with our cohort, previous studies (29–31) either performed desensitization at a lower frequency or did not report it, used induction therapy less frequently, or used cyclosporine as the main maintenance immunosuppressive agent more frequently. Taken together, the results indicate that the use of a more robust immunosuppressive protocol in our cohort may explain the lower incidence of ABMR. However, despite an overall decrease in the incidence of BPAR, patients with HLA-DSA experienced ABMR more frequently than those without HLA-DSA, whereas no such difference was observed in the development of TCMR. These results are consistent with those of previous reports (29–31, 33) and suggest that XM-negative HLA-DSA affects the development of ABMR.

We further investigated which HLA-DSA characteristics—number, class, or strength—specifically increased the likelihood of ABMR. The incidence of ABMR tended to be higher in patients with multiple HLA-DSAs compared to those with a single HLA-DSA. Preformed alloantibodies are known to cause acute ABMR predominantly through complement cascade activation and, to a lesser extent, through complement-independent mechanisms (34). Anti-HLA antibodies are the most representative biomarkers for predicting ABMR (2) and are the diagnostic criteria for ABMR (35). The presence of multiple HLA-DSAs suggests a broader immune response against the allograft kidney, amplifying the risk of ABMR as each HLA-DSA targets specific antigens. In addition, patients with both class I and II HLA-DSAs tended to exhibit a higher incidence of ABMR than those with class I or II HLA-DSA alone, consistent with the findings of previous reports (36–38). Class I HLA-DSA induces endothelial damage through complement activation, whereas class II HLA-DSA binds to antigen-presenting cells, promoting T-cell activation and interaction with B cells (39). These distinct mechanisms may increase the risk of ABMR and have a synergistic effect in patients with both class I and II HLA-DSA. When stratified by HLA-DSA MFI values, patients with moderate HLA-DSA exhibited a higher frequency of ABMR than those with weak HLA-DSA. The MFI value is commonly used to indicate the strength of HLA-DSA, largely because high MFI values correlate with increased complement-binding capacity and more severe tissue damage (2). Consistent with this, many previous clinical studies have reported that the risk of ABMR increases with higher HLA-DSA MFI values (6, 37, 38, 40). However, this trend was inconsistent in patients with strong HLA-DSA, which may be attributable to the extremely small sample size (only one ABMR event in ten patients). Notably, strong HLA-DSA primarily comprises anti-HLA-DQ and anti-HLA-Cw antibodies; however, the clinical significance of pre-transplant anti-HLA-DQ and anti-HLA-Cw antibodies remains uncertain (41). Consequently, the unexpectedly low incidence of ABMR may possibly due to the specificity of strong HLA-DSA. Furthermore, in the ROC analysis using MFI^peak^ and MFI^sum^ values of HLA-DSA, the AUC value was 0.622 (95% CI: 0.427–0.818) for MFI^peak^ and 0.622 (95% CI: 0.425–0.820) for MFI^sum^. Therefore, it was not possible to predict the development of ABMR based solely on MFI values. Given the very low number of patients and ABMR events in subgroups categorized by the number, class, and strength of HLA-DSA, these findings should be interpreted with utmost caution.

We subsequently examined long-term clinical outcomes. The presence of pre-transplant HLA-DSA increased the risk of ABMR but did not affect kidney allograft function and survival outcomes, including allograft, patient, and infection-free survival. PP, with or without a combination of IVIG and glucocorticoids, is the standard treatment for patients with ABMR (42). To reduce antibody production, the use of agents such as RTX (an anti-CD20 monoclonal antibody) and bortezomib has been introduced and is now actively employed in ABMR management (34). In our cohort, the patients diagnosed with ABMR were treated with various combination therapies, including RTX and bortezomib. In our cohort, 13 of 237 (5.5%) patients in the HLA-DSA (+) group developed ABMR, with only one experiencing death-censored graft failure. In contrast, 27 of 1782 (1.5%) patients in the HLA-DSA (-) group developed ABMR, and three of these experienced death-censored allograft failure. This indicates that ABMR itself, rather than the presence of pre-transplant XM-negative HLA-DSA, significantly impacts allograft survival. Unfortunately, we could not confirm the HLA-DSA status at the time of ABMR diagnosis for the 27 patients in the HLA-DSA (-) group because our cohort has been collecting data of post-transplant HLA-DSA only since 2017. However, it is likely that *de novo* HLA-DSAs contributed to the ABMR in these cases. Previous studies have shown that ABMR resulting from pre-transplant HLA-DSA exhibits better graft survival compared to ABMR caused by *de novo* HLA-DSA under intensive immunosuppression (43). Therefore, the favorable graft survival observed in the HLA-DSA (+) group in our study might be attributed to effective control of ABMR through pre-transplant strong immunosuppression and aggressive therapeutic interventions. Previous studies on the clinical impact of XM-negative HLA-DSA have included diverse proportions of deceased donor KT and LDKT (10, 11). In a large-scale retrospective study conducted by Orandi et al. (26), only LDKT recipients were analyzed, and their findings suggested that XM-negative HLA-DSA had no impact on the 5-year graft survival. Moreover, in studies comparing clinical outcomes according to the donor type, the impact of XM-negative HLA-DSA was more pronounced in DDKT than in LDKT (12, 44). Thus, by focusing exclusively on LDKT in this study, we likely observed a more favorable long-term outcome compared to previous studies.

In most studies on pre-transplant XM-negative HLA-DSA, desensitization was either not performed or unreported (10, 11). In a single-center study conducted in Japan (45), patients with XM-negative HLA-DSA experienced significantly higher rates of acute and chronic rejection than those without HLA-DSA. However, following the implementation of a desensitization protocol using double-filtration PP and RTX, there was a significant reduction in rejection events and improvement of 5-year graft survival rates. In our cohort, although we expected patients with pre-transplant HLA-DSA who underwent desensitization to experience fewer ABMR events, the findings were contrary to our expectations. The desensitized group exhibited higher class I and class II PRA levels and higher HLA-DSA MFI values than the non-desensitized group. In other words, desensitization was selectively performed in more sensitized patients with stronger HLA-DSA. Therefore, the subgroup analysis had a strong selection bias. To address this concern, we performed a stratified analysis based on HLA-DSA MFI values. However, in each stratified group, a consistent trend of the desensitized group exhibiting a higher incidence of ABMR irrespective of MFI values was observed. Furthermore, the limited sample size and a significant number of missing values prevented us from using propensity score method. Thus, we could not determine whether desensitization reduced the risk of ABMR in patients with XM-negative HLA-DSA. Additionally, the infection-free survival rate tended to be lower in the desensitized group compared to the non-desensitized group. To validate the effects of desensitization on ABMR development and post-transplant infection in patients with pre-transplant XM-negative HLA-DSA, prospective studies employing consistent desensitization protocols are needed.

This study had some limitations. A mean follow-up period of 20.5 months may not be sufficient to estimate long-term allograft outcomes. Moreover, there was no standardized desensitization protocol for pre-transplant XM-negative HLA-DSA, and the decision to perform desensitization and the methods used varied among transplant centers. These differences could lead to a potential overestimation or underestimation of the clinical impact of HLA-DSA. Lastly, our data lacked detailed information on HLA-DSA characteristics, such as complement binding affinity (e.g., C1q or C4d assay), immunoglobulin G subclasses, and HLA eplet mismatches. Nonetheless, a strength of the present study is its nationwide multicenter design, encompassing a significant number of LDKT recipients with pre-transplant XM-negative HLA-DSA compared with previous studies.

In conclusion, pre-transplant XM-negative HLA-DSA increased the risk of ABMR but did not affect long-term allograft outcomes. A more detailed and standardized desensitization protocol that considers HLA-DSA characteristics, such as number, class, and intensity, is needed. Under the current regimen of standardized immunosuppressive induction, maintenance, and appropriate desensitization, HLA-incompatible KT appears to be feasible in the context of XM-negative HLA-DSA. Nonetheless, careful monitoring and timely intervention are essential for any ABMR event.

## Data availability statement

The original contributions presented in the study are included in the article/**Supplementary Material**, further inquiries can be directed to the corresponding author.

## Ethics statement

This study received approval from the Institutional Review Board of Seoul St. Mary’s Hospital (KC14ONMI0460). The studies were conducted in accordance with the local legislation and institutional requirements. The ethics committee/institutional review board waived the requirement of written informed consent for participation from the participants or the participants’ legal guardians/next of kin because of the retrospective study design and the use of noninvasive procedures.

## Author contributions

HL: Conceptualization, Data curation, Methodology, Writing – original draft, Formal analysis, Investigation, Visualization. HBL: Conceptualization, Writing – review & editing. IS: Writing – review & editing. JP: Writing – review & editing. J-WP: Writing – review & editing. TB: Writing – review & editing. JY: Writing – review & editing. MK: Writing – review & editing. CY: Writing – review & editing. BC: Conceptualization, Funding acquisition, Supervision, Writing – review & editing.
