# Corrigendum: Pre-transplant crossmatch-negative donor-specific anti-HLA antibody predicts acute antibody-mediated rejection but not long-term outcomes in kidney transplantation: an analysis of the Korean Organ Transplantation Registry

**DOI:** 10.3389/fimmu.2024.1467474

**Published:** 2024-08-01

**Authors:** Haeun Lee, Hanbi Lee, In O Sun, Jung Hwan Park, Jong-Won Park, Tae Hyun Ban, Jaeseok Yang, Myoung Soo Kim, Chul Woo Yang, Byung Ha Chung, Korean Organ Transplantation Registry Study Group

**Affiliations:** ^1^ Division of Nephrology, Department of Internal Medicine, Presbyterian Medical Center, Jeonju, Republic of Korea; ^2^ Division of Nephrology, Department of Internal Medicine, Seoul St. Mary’s Hospital, The Catholic University of Korea, Seoul, Republic of Korea; ^3^ Department of Nephrology, Konkuk University School of Medicine, Seoul, Republic of Korea; ^4^ Department of Nephrology, Yeungnam University Hospital, Daegu, Republic of Korea; ^5^ Division of Nephrology, Department of Internal Medicine, Eunpyeong St. Mary’s Hospital, The Catholic University of Korea, Seoul, Republic of Korea; ^6^ Division of Nephrology, Department of Internal Medicine, Yonsei University College of Medicine, Severance Hospital, Seoul, Republic of Korea; ^7^ Department of Surgery, Yonsei University College of Medicine, Seoul, Republic of Korea

**Keywords:** kidney transplantation, donor-specific anti-HLA antibody, solid phase assay, rejection, antibody-mediated rejection, desensitization

In the published article, there was an error in affiliation 2. Instead of “Division of Nephrology, Department of Internal Medicine, Seoul St. Mary’s Hospital, Seoul, Republic of Korea”, it should be “Division of Nephrology, Department of Internal Medicine, Seoul St. Mary’s Hospital, The Catholic University of Korea, Seoul, Republic of Korea”.

In the published article, there was an error in affiliation 5. Instead of “Division of Nephrology, Department of Internal Medicine, Eunpyeong St. Mary’s Hospital, Seoul, Republic of Korea” it should be “Division of Nephrology, Department of Internal Medicine, Eunpyeong St. Mary’s Hospital, The Catholic University of Korea, Seoul, Republic of Korea”.

The authors apologize for this error and state that this does not change the scientific conclusions of the article in any way. The original article has been updated.

