# Supplementary material for: Pre-transplant crossmatch-negative donor-specific anti-HLA antibody predicts acute antibody-mediated rejection but not long-term outcomes in kidney transplantation: an analysis of the Korean Organ Transplantation Registry
Source: Front Immunol. 2024 Jul 11;15:1420351. doi: 10.3389/fimmu.2024.1420351 (PMC11269232; doi:10.3389/fimmu.2024.1420351)
Supplement: Supplementary file 1 [file Presentation_1.pptx]

## Slide 1
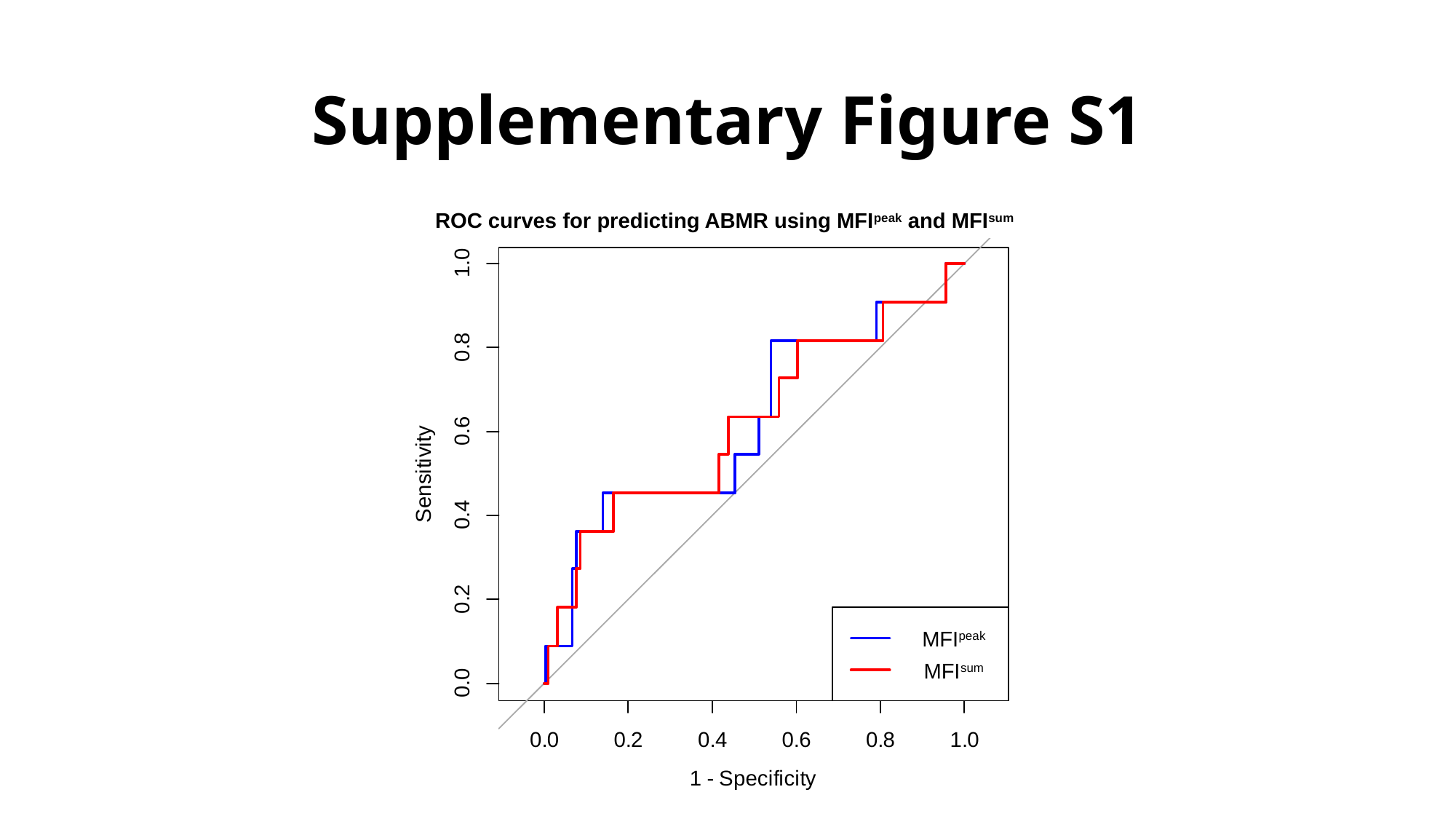

# Supplementary Figure S1
ROC curves for predicting ABMR using MFIpeak and MFIsum
MFIpeak
MFIsum

## Slide 2
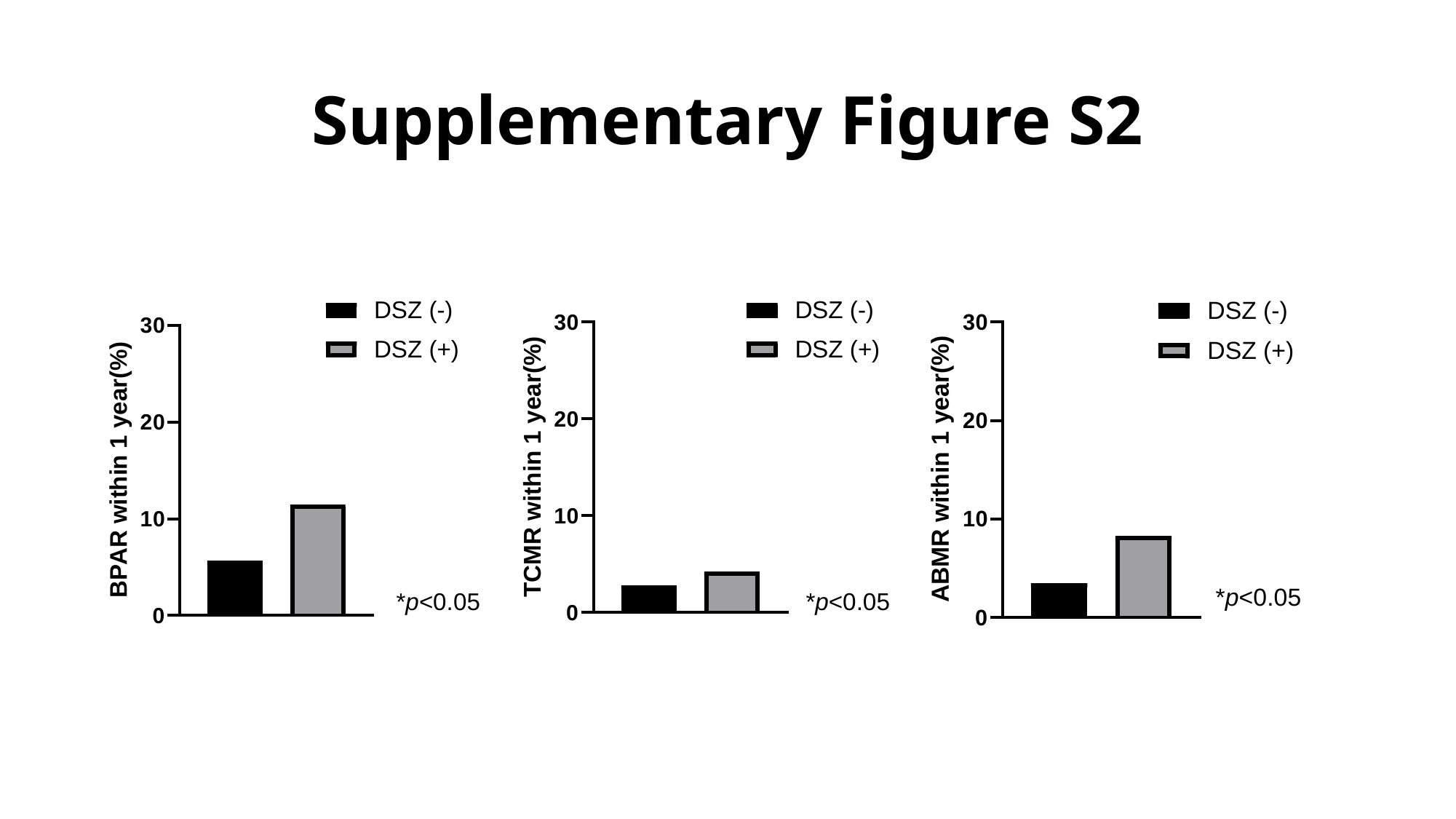

# Supplementary Figure S2

## Slide 3
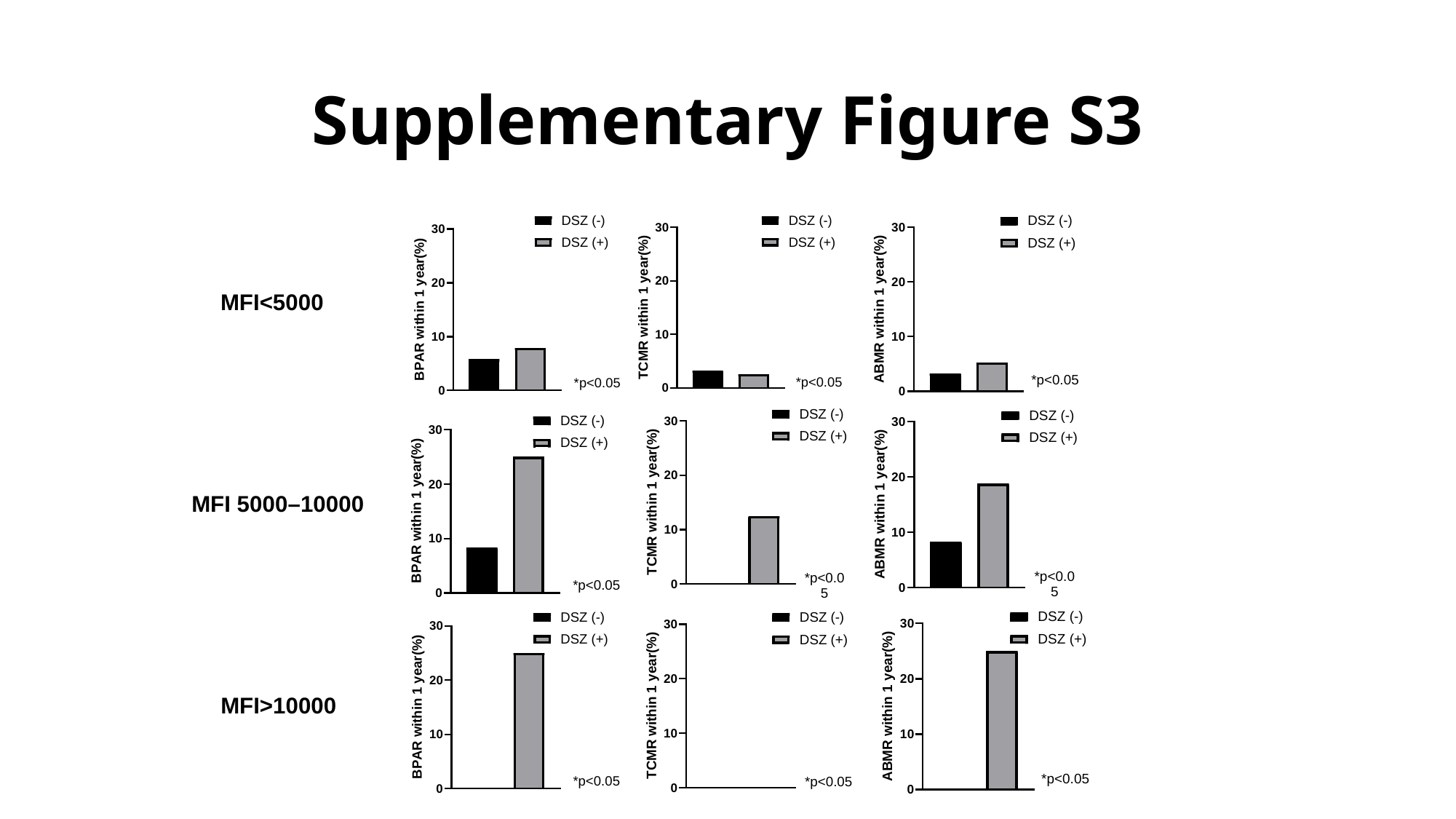

Supplementary Figure S3
MFI<5000
MFI 5000–10000
MFI>10000

## Slide 4
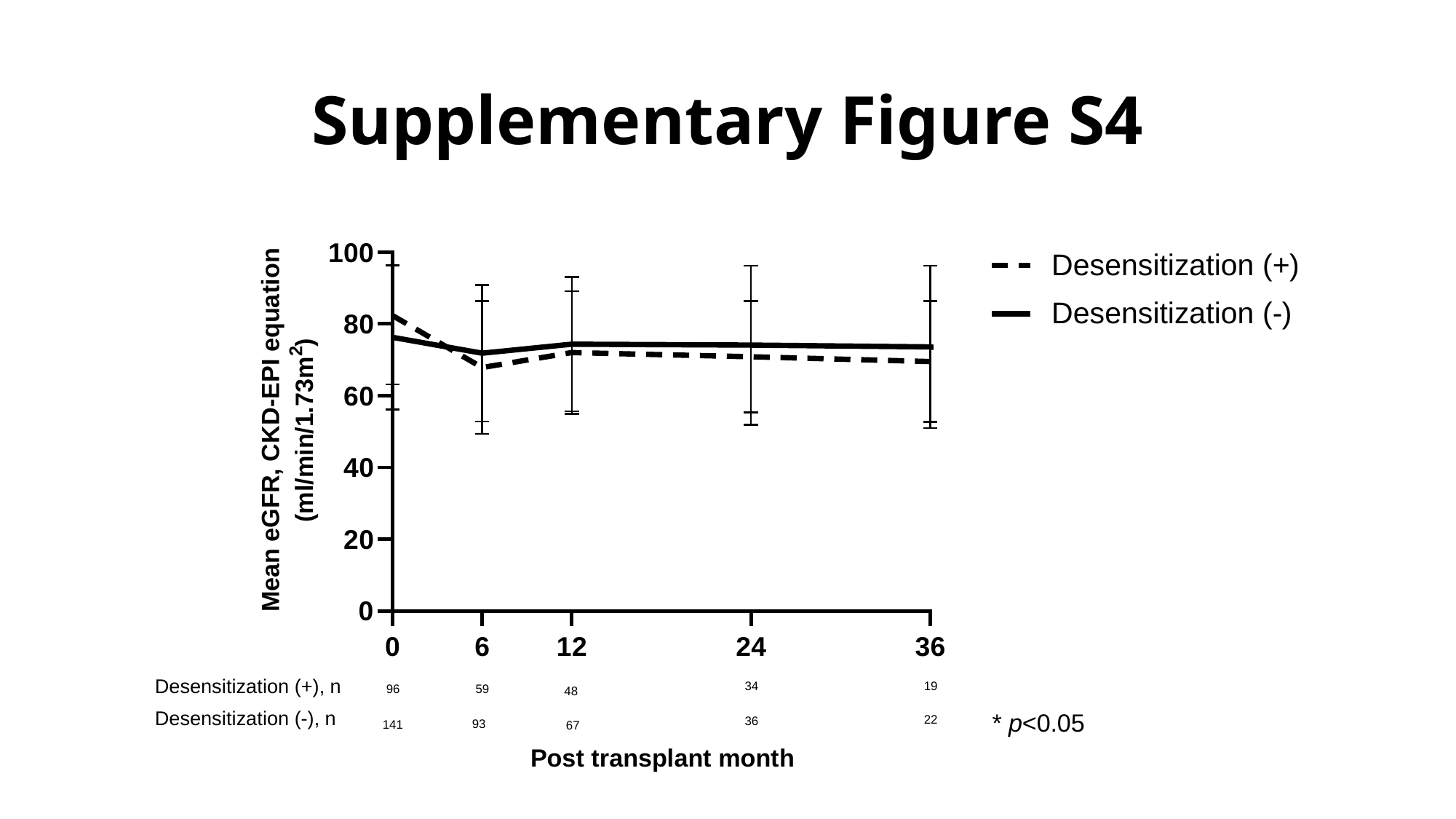

# Supplementary Figure S4

## Slide 5
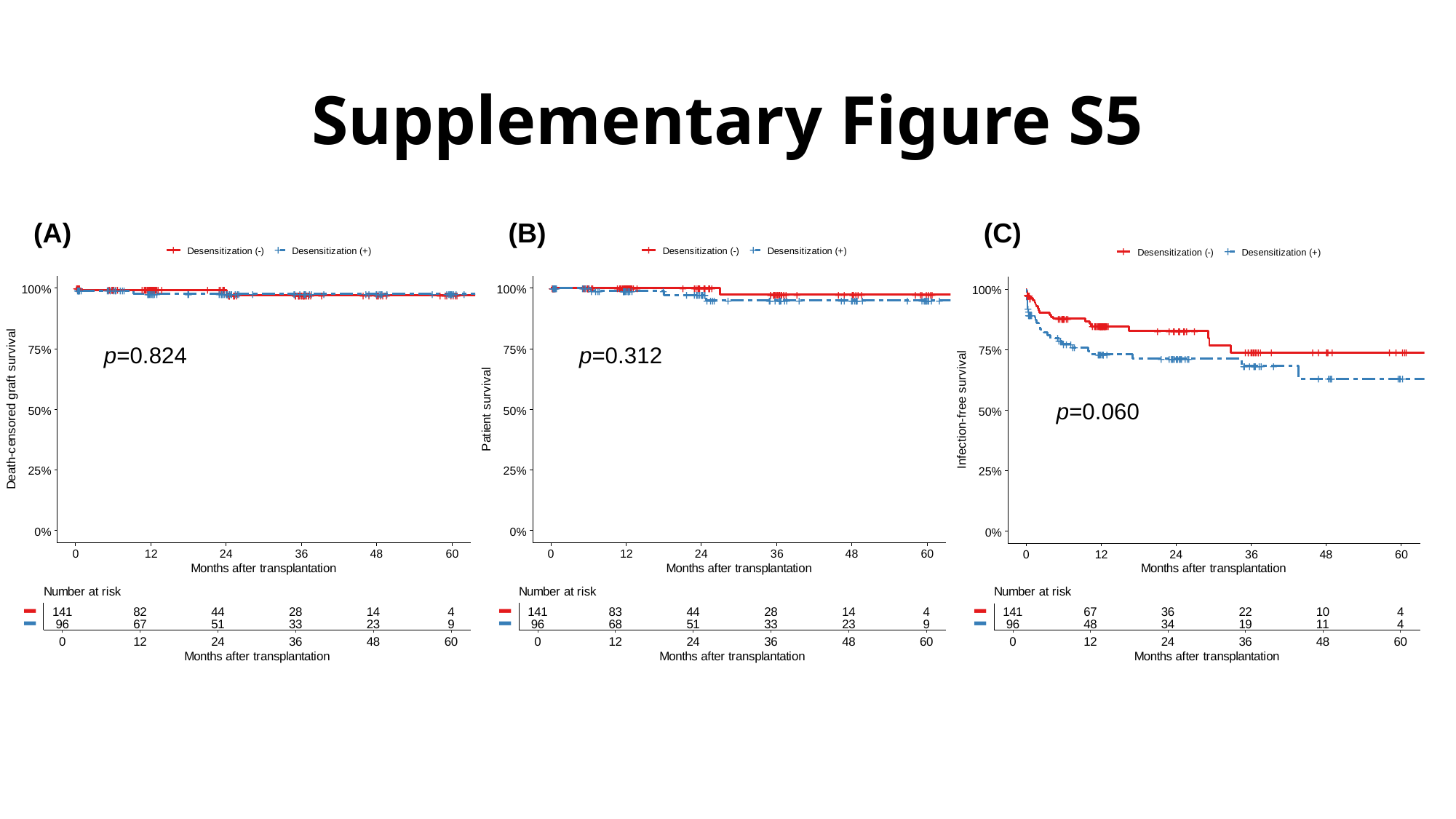

# Supplementary Figure S5
(A)
(B)
(C)
p=0.824
p=0.312
p=0.060
