# Supplementary material for: Pre-transplant crossmatch-negative donor-specific anti-HLA antibody predicts acute antibody-mediated rejection but not long-term outcomes in kidney transplantation: an analysis of the Korean Organ Transplantation Registry
Source: Front Immunol. 2024 Jul 11;15:1420351. doi: 10.3389/fimmu.2024.1420351 (PMC11269232; doi:10.3389/fimmu.2024.1420351)
Supplement: Supplementary file 2 [file Table_1.docx]

**Supplementary Table S1. Correlation between ABMR and death-censored graft failure.**

| No. | HLA-DSA | ABMR treatment | | Response to treatment | Death-censored graft failure | | Cause of graft failure | Time from ABMR to death-censored graft failure (months) |
| --- | --- | --- | --- | --- | --- | --- | --- | --- |
| 1 | (+) | Steroid pulse+PP+IVIG | Resolution with allograft dysfunction, Cr < 2.8mg/dL | | |  |  |  |
| 2 | (+) | Steroid pulse | Resolution with allograft dysfunction, Cr < 2.8mg/dL | | |  |  |  |
| 3 | (+) | Observation | Resolution with stabilized allograft function | | |  |  |  |
| 4 | (+) | PP | Inadequate control with graft failure within 1 month | | | Yes | Rejection | 0.1 |
| 5 | (+) | Steroid pulse+PP+IVIG+RTX | Resolution with stabilized allograft function | | |  |  |  |
| 6 | (+) | Steroid pulse+PP+IVIG+RTX | Resolution with stabilized allograft function | | |  |  |  |
| 7 | (+) | Observation | Resolution with stabilized allograft function | | |  |  |  |
| 8 | (+) | Observation | Resolution with stabilized allograft function | | |  |  |  |
| 9 | (+) | Steroid pulse+ATG+PP+IVIG+RTX | Resolution with stabilized allograft function | | |  |  |  |
| 10 | (+) | Observation | Resolution with allograft dysfunction, Cr < 2.8mg/dL | | |  |  |  |
| 11 | (+) | Steroid pulse+PP+IVIG | Resolution with stabilized allograft function | | |  |  |  |
| 12 | (+) | Steroid pulse | Resolution with allograft dysfunction, Cr < 2.8mg/dL | | |  |  |  |
| 13 | (+) | Steroid pulse+ATG | Resolution with stabilized allograft function | | |  |  |  |
| 14 | (-) | IST adjustment+Steroid pulse | Resolution with stabilized allograft function | | |  |  |  |
| 15 | (-) | Steroid pulse+ATG+PP+IVIG | Resolution with stabilized allograft function | | |  |  |  |
| 16 | (-) | Steroid pulse+PP | Resolution with stabilized allograft function | | |  |  |  |
| 17 | (-) | Steroid pulse+PP+IVIG+RTX | Resolution with stabilized allograft function | | |  |  |  |
| 18 | (-) | PP+IVIG | Resolution with stabilized allograft function | | |  |  |  |
| 19 | (-) | IST adjustment+Steroid pulse+ATG+PP | Resolution with stabilized allograft function | | |  |  |  |
| 20 | (-) | Steroid pulse+ATG+PP+IVIG+RTX | Resolution with allograft dysfunction, Cr < 2.8mg/dL | | |  |  |  |
| 21 | (-) | Steroid pulse+PP+IVIG+RTX | Resolution with stabilized allograft function | | |  |  |  |
| 22 | (-) | Steroid pulse+ATG+PP+IVIG+RTX | Resolution with allograft dysfunction, Cr ≥ 2.8mg/dL | | | Yes | Unknown | 20.2 |
| 23 | (-) | Steroid pulse+RTX+IVIG | Resolution with stabilized allograft function | | |  |  |  |
| 24 | (-) | Steroid pulse+PP+IVIG+RTX | Resolution with stabilized allograft function | | |  |  |  |
| 25 | (-) | Steroid pulse+PP+IVIG+RTX | Resolution with allograft dysfunction, Cr < 2.8mg/dL | | |  |  |  |
| 26 | (-) | Steroid pulse+ATG+PP+IVIG+RTX+BTZ | Resolution with stabilized allograft function | | |  |  |  |
| 27 | (-) | Steroid pulse+PP+IVIG+RTX | Resolution with stabilized allograft function | | |  |  |  |
| 28 | (-) | Steroid pulse | Resolution with stabilized allograft function | | |  |  |  |
| 29 | (-) | Steroid pulse+PP+IVIG+RTX | Resolution with stabilized allograft function | | |  |  |  |
| 30 | (-) | Steroid pulse | Resolution with allograft dysfunction, Cr < 2.8mg/dL | | |  |  |  |
| 31 | (-) | Observation | Resolution with stabilized allograft function | | |  |  |  |
| 32 | (-) | ATG+PP+IVIG+RTX | Resolution with stabilized allograft function | | |  |  |  |
| 33 | (-) | IST adjustment+Steroid pulse | Resolution with stabilized allograft function | | | Yes | Rejection | 14.0 |
| 34 | (-) | Steroid pulse+PP+IVIG+RTX | Resolution with stabilized allograft function | | |  |  |  |
| 35 | (-) | IST adjustment+Steroid pulse+ATG+PP+RTX | Resolution with stabilized allograft function | | |  |  |  |
| 36 | (-) | Steroid pulse+ATG+PP+RTX | Resolution with allograft dysfunction, Cr < 2.8mg/dL | | | Yes | Rejection | 10.8 |
| 37 | (-) | Steroid pulse | Resolution with stabilized allograft function | | |  |  |  |
| 38 | (-) | Steroid pulse+PP+RTX | Resolution with stabilized allograft function | | |  |  |  |
| 39 | (-) | Steroid pulse+PP+IVIG+RTX | Resolution with allograft dysfunction, Cr ≥ 2.8mg/dL | | |  |  |  |
| 40 | (-) | Steroid pulse | Resolution with allograft dysfunction, Cr ≥ 2.8mg/dL | | |  |  |  |

ABMR, antibody-mediated rejection; BTZ, bortezomib; Cr, serum creatinine; IST, immunosuppressant; IVIG, intravenous immunoglobulin; HLA-DSA, donor-specific anti-human leukocyte antigen antibody; PP, plasmapheresis; RTX, rituximab.
