# Supplementary material for: Pre-transplant crossmatch-negative donor-specific anti-HLA antibody predicts acute antibody-mediated rejection but not long-term outcomes in kidney transplantation: an analysis of the Korean Organ Transplantation Registry
Source: Front Immunol. 2024 Jul 11;15:1420351. doi: 10.3389/fimmu.2024.1420351 (PMC11269232; doi:10.3389/fimmu.2024.1420351)
Supplement: Supplementary file 3 [file Table_2.docx]

**Supplementary Table S2. Baseline characteristics of patients with pre-transplant HLA-DSA according to desensitization status**

|  | **Desensitization (+)**  **(n=96)** | **Desensitization (-)**  **(n=141)** | ***p*-value** |
| --- | --- | --- | --- |
| **Recipient** |  |  |  |
| Age (years) | 50.3 ± 10.8 | 49.1 ± 12.0 | 0.532 |
| Female sex (n, %) | 68 (70.8%) | 77 (54.6%) | 0.017 |
| Primary renal disease (n, %) |  |  | 0.257 |
| Diabetes | 22 (22.9%) | 36 (25.5%) |  |
| Hypertension | 9 (9.4%) | 20 (14.2%) |  |
| Glomerulonephritis | 37 (38.5%) | 37 (26.2%) |  |
| Polycystic kidney disease | 8 (8.3%) | 10 (7.1%) |  |
| Others | 3 (3.1%) | 2 (1.4%) |  |
| Unknown | 17 (17.7%) | 36 (25.5%) |  |
| BMI | 22.7 ± 3.6 | 23.3 ± 3.8 | 0.175 |
| PRA class I (%) | 34.0 (1.5 – 61.0) | 11.0 (0 - 36.0) | 0.038 |
| PRA class II (%) | 32.5 (0.0 – 57.0) | 15.0 (0.0 – 34.0) | 0.028 |
| Dialysis vintage (months) | 26.6 ± 54.1 | 29.2 ± 55.1 | 0.134 |
| Follow up duration (months) | 27.7 ± 21.2 | 18.8 ± 17.1 | 0.001 |
| **Donor** |  |  |  |
| Age (years) | 46.3 ± 12.2 | 47.3 ± 12.2 | 0.452 |
| Female sex (n, %) | 48 (50.0%) | 67 (47.5%) | 0.808 |
| BMI | 23.9 ± 3.2 | 24.4 ± 3.1 | 0.394 |
| **HLA-DSA characteristics** |  |  |  |
| Strength (MFI^peak^) | 2670 (1730–4520) | 1970 (1320–3480) | 0.007 |
| MFI^peak^, group |  |  | 0.197 |
| Weak (<5000) | 76 (79.2%) | 121 (85.8%) |  |
| Moderate (5000–10000) | 16 (16.7%) | 12 (8.5%) |  |
| Strong (>10000) | 4 (4.2%) | 6 (4.3%) |  |
| Strength (MFI^sum^) | 3270 (1910–4990) | 2100 (1330–4230) | 0.002 |
| Class |  |  | 0.626 |
| Class I | 37 (38.5%) | 62 (44.0%) |  |
| Class II | 45 (46.9%) | 63 (44.7%) |  |
| Class I + II | 14 (14.6%) | 16 (11.3%) |  |
| Number |  |  | 0.575 |
| 1 | 67 (69.8%) | 108 (76.6%) |  |
| 2 | 23 (24.0%) | 27 (19.1%) |  |
| 3 | 5 (5.2%) | 4 (2.8%) |  |
| 4 | 1 (1.0%) | 2 (1.4%) |  |
| **Transplant** |  |  |  |
| Re-transplant (n, %) | 11 (11.5%) | 12 (8.5%) | 0.597 |
| Cold ischemic time | 56.9 ± 32.8 | 56.0 ± 34.5 | 0.698 |
| Induction therapy (n, %) |  |  | 0.003 |
| No | 0 (0%) | 2 (1.4%) |  |
| ATG | 43 (44.8%) | 48 (34.0%) |  |
| Basiliximab | 46 (47.9%) | 90 (63.8%) |  |
| ATG and Basiliximab | 7 (7.3%) | 1 (0.7%) |  |
| Maintenance immunosuppressant (n, %) |  |  | 0.147 |
| Tacrolimus | 95 (99.0%) | 133 (94.3%) |  |
| Cyclosporine | 1 (1.0%) | 7 (5.0%) |  |
| Tacrolimus and Sirolimus | 0 (0%) | 1 (0.7%) |  |

ATG, anti-thymocyte globulin; BMI, body mass index; HLA-DSA, donor-specific anti-human leukocyte antigen antibody; MFI, mean fluorescence intensity; MFI^peak^, peak MFI; MFI^sum^, total MFI of all HLA-DSAs; PRA, panel-reactive antibody.
